# Anemia design effects in cluster surveys of women and young children in refugee settings

**DOI:** 10.1371/journal.pone.0254031

**Published:** 2021-07-15

**Authors:** Erin N. Hulland, Eva Leidman, Caroline Wilkinson, Mélody Tondeur, Oleg Bilukha

**Affiliations:** 1 Emergency Response and Recovery Branch, Division of Global Health Protection, Centers for Disease Control and Prevention, Atlanta, Georgia, United States of America; 2 Division of Programme Support and Management, Public Health Section, United Nations High Commissioner for Refugees, Geneva, Switzerland; 3 Canadian Partnership for Women and Children’s Health, Peterborough, Ontario, Canada; University of Washington, UNITED STATES

## Abstract

**Background:**

Nutrition surveys in many refugee settings routinely estimate anemia prevalence in high-risk population groups. Given the lack of information on anemia design effects (DEFF) observed in surveys in these settings, the goal of this paper is to better understand the magnitude and distribution of DEFFs and intracluster correlation coefficients (ICCs) in order to inform future survey design.

**Methods:**

Two-stage cluster surveys conducted during 2013–2016 were included if they measured hemoglobin in refugee children aged 6–59 months and/or non-pregnant women aged 15–49 years. Prevalence of anemia, anemia DEFFs and ICCs, mean cluster size, number of clusters, and total sample size were calculated per-survey for non-pregnant women and children. Non-parametric tests were used to assess differences and correlations of ICC and DEFF between women and children and inter-regional differences.

**Results:**

Eighty-seven unique cluster surveys from nine countries were included in this analysis. More than 90% of all surveys had ICC values for anemia below 0.10. Median ICC for children was 0.032 (IQR: 0.015–0.048), not significantly different from that observed for non-pregnant women for whom the median was 0.024 (IQR: -0.002–0.055). DEFFs were significantly higher for children [1.54 (IQR: 1.21–1.82)] versus women [1.20 (IQR: 0.99–1.46)]. Regional differences in DEFFs and ICCs were observed.

**Conclusions:**

Both ICCs and DEFF were relatively small for both non-pregnant women and preschool children and fall in a narrow range. Differences in ICCs between women and children were non-significant, suggesting similar inter-cluster distributions of anemia; significant differences in DEFF were likely attributable to differing cluster sizes. Given regional differences in both ICCs and DEFFs, location-specific values are preferred. However, in the absence of other context-specific information, we suggest using DEFFs of 1.4–1.8 if mean cluster size is around 20, and DEFFs of 1.2–1.4 if mean cluster size is around 10.

## Introduction

Anemia affects an estimated 1.7 billion people worldwide, with the bulk of the infections occurring among women and young children in Sub-Saharan Africa and South Asia [1]. Most commonly attributable to micronutrient deficiencies, anemia is associated with poor birth outcomes, maternal mortality, developmental delays, and disability [2, 3]. A recent study estimated that globally two in every five children aged 6 to 59 months were anemic (39.7% prevalence) and more than one in every five persons globally was anemic (22.8% prevalence) [1]. Due to the high burden of anemia among women of reproductive age and the associated sequelae, anemia reduction was endorsed by the 2012 World Health Assembly as one of the six global nutrition targets for 2025 as detailed in the “Comprehensive implementation plan on maternal, infant, and young child nutrition” [4].

Although prevalent globally, anemia is a particular concern in refugee settings where risk factors including restricted diets, increased transmission of infectious diseases, malaria, cramped living conditions, and inadequate access to water and sanitation may be present [5]. Anemia may be a concern even where acute malnutrition is not, as documented in recent surveys of Syrian refugees in Lebanon and Jordan [6]. Hemoglobin is often measured during patient consultations at health facilities in most refugee camps for individual diagnostic purposes. However, representative population surveys are considered the gold standard for estimating prevalence of anemia for public health action. Surveys allow humanitarian organizations to quantify the burden of anemia among women and children at the population level. As such, they are particularly important as an indication of micronutrient status especially in contexts where food assistance has been reduced and fortified foods are not available for women or children and in protracted emergency settings where refugees are often dependent on humanitarian assistance as the primary source of foods for years.

Measurement of hemoglobin as part of household surveys in refugee camps has only recently become routine practice. Most population-representative household surveys focused on nutritional status in refugee settings historically collected measures of acute malnutrition rather than micronutrient deficiencies, in part due to feasibility of measuring anthropometry in households relative to other metrics requiring laboratory testing [7]. With the release of the first HemoCue in 1999, measuring hemoglobin concentration in household surveys became much simpler, requiring only a few drops of capillary blood (via a fingertip prick) that could be tested immediately using a small, durable, and reasonably affordable test. The rapid test eliminated barriers related to proper storage and transport of blood samples to a laboratory that previously made testing of hemoglobin prohibitively complicated, particularly in humanitarian settings. In 2011, the United Nations High Commissioner for Refugees (UNHCR) released the first version of the survey methodology specific to conducting surveys of refugee populations [8–10]. This methodology, Standardized Expanded Nutrition Survey (SENS), recommended the inclusion of anemia where programmatically appropriate and provided detailed guidelines for how to measure anthropometry and anemia, as well as select additional groups of indicators of priority in refugee settings. In parallel to the release of these standardized survey guidelines, UNHCR Public Health Section developed a central, global database to track key survey results obtained, and store all raw data collected and final reports produced.

As part of this guidance, UNHCR recommended customizing sample size calculations specific to each context using a standard simple proportion sample size calculation and a Student’s t-score with degrees of freedom equal to the number of clusters minus 1 [11]. Design Effects (DEFF) are used in sample size calculations for cluster surveys as an adjustment factor to account for the loss of precision resulting from sampling within clusters, and consequently within-cluster homogeneity. As field collection of hemoglobin data remains infrequent, literature on design parameters for anemia is sparse [12]. Previous research has demonstrated that DEFFs for different indicators vary considerably given differences in clustering in time and space. For example, one study found that programmatic indicators such as access to Vitamin A supplementation had DEFFs >10 whereas DEFFs for anthropometric indicators were generally within the range of 1 and 2 for surveys with the same sampling design [13]. A recent meta-analysis of 380 surveys from 28 countries found that DEFF for stunting (median = 1.77) was higher than wasting (median = 1.35) even after adjusting for prevalence and mean cluster size [14]. Additional empirical data describing the distribution of hemoglobin is therefore useful to inform design of all cluster surveys estimating anemia, including SENS surveys.

Ten years since the release of the initial SENS guidance, there is now a sufficiently large body of evidence to allow for multi-country analysis of cluster field nutrition surveys including hemoglobin measurements. To inform survey design, this manuscript aims to describe the magnitude and distribution of anemia DEFFs and corresponding ICCs, as well as other variables related to survey design and implementation.

## Methods

### Data collection and processing

Data for these analyses were obtained from population representative cluster surveys conducted by UNHCR and partners in refugee settings between 2013 and 2016 that applied the SENS methodology [9]. Surveys were sub-national, conducted in populations of concern to UNHCR, defined as refugee camps and small communities hosting refugees [15]. All included surveys measured hemoglobin in non-pregnant women aged 15–49 years, children aged 6–59 months, or both target groups. Surveys also collected anthropometric data (e.g., weight, height/length, and age) of children aged 6–59 months and select other variables depending on programmatic needs. All surveys used a two-stage cluster sampling methodology. Probability proportional to size was used to sample clusters at stage one and households were selected using random methods at stage two [9, 10]. Anthropometric calculations are described in depth elsewhere [14].

Following SENS guidelines, surveyors measured hemoglobin of women in every other selected household and measured hemoglobin of children aged 6–59 months at either every selected household, or every other selected household, depending on whether or not public health interventions to address anemia were planned or ongoing for the survey area. Hemoglobin concentration in capillary (fingertip prick) blood samples were measured in grams per deciliter (g/dL) or grams per liter (g/L) using the HemoCue^®^ Hb 301 Analyser [7, 8]. Total anemia was defined as hemoglobin measures less than 12 g/dL for women and less than 11 g/dL for children [16]. Biologically implausible hemoglobin values, greater than 17 g/dL or less than 4 g/dL, were excluded from analysis [17]. Information not available in standardized data sets were obtained from camp-specific survey reports in the UNHCR global database. This study constitutes a secondary analysis of survey data collected for programmatic purposes. Informed verbal consent was obtained from each survey participant by UNHCR and partners individually for each survey.

### Data analysis

All analyses were conducted at the survey-level. At the individual level during summarization, children without age, sex, or hemoglobin measurements were excluded as were women without pregnancy status or hemoglobin data. At the survey-level, surveys in which ≥70% of respondents were missing hemoglobin data were excluded from the analysis. From each survey, we extracted survey year and country, as well as mean cluster size, total sample size, number of clusters, anemia prevalence, standard deviation of hemoglobin values, and anemia DEFFs for both women and children.

DEFFs were estimated from survey-level data using the Taylor series variance estimation (a linearization method) for simplicity and interpretability [11, 18]. ICC is a measure of within- and between-cluster variability independent of cluster size, and thus is better suited for cross-population comparisons where mean cluster sizes vary across surveys. We calculated ICCs using the estimated DEFFs and mean cluster size (Formula 1). Due to the fact that Taylor series approximation procedure in rare instances can produce a DEFF estimate that is below 1, applying Formula 1 produces negative ICCs in those instances, which is sometimes the case with highly variable outcomes and small sample sizes [19].


DEFF=1+ICC*(MeanClusterSize-1) (1)

Distributions of DEFFs and related covariates were assessed across all eligible surveys, and the percentage of surveys with DEFF below the commonly used thresholds of design effects– 1.50, 1.75, and 2.00 –were calculated. We also calculated the percentage of ICCs under 0.02 –equivalent to a DEFF of 1.50 using a mean cluster size of 25 –and two other ICC thresholds: 0.05 and 0.10. Shapiro-Wilk tests and QQ plots were used to assess normality of the DEFFs and ICCs. Wilcoxon signed-rank tests were run to assess survey-level differences in DEFFs, ICCs, and mean cluster size between surveys of women and children. For surveys which measured hemoglobin among both women and children from sampled households, correlations of survey DEFFs between women and children and of survey ICCs between women and children were assessed using Spearman’s correlations to account for non-normality of the data. Kruskal-Wallis tests looking at DEFFs and ICCs as our outcomes of interest, using Bonferroni’s adjustments for post-hoc analyses, were assessed overall across all regional groupings and between all pairwise comparisons of regions–Kenya vs. Chad, Chad vs Other, Kenya vs. Other–to assess any need for differing guidance by country. All presented analysis were performed using SAS version 9.3 and R version 3.5.0; figures were created using ggplot2 [20–25].

## Results

Overall, 88 surveys conducted between 2013 and 2016 were obtained from UNHCR (Table 1). One survey was excluded from the analysis because of a high proportion of missing hemoglobin measurements. The median percentage of women missing hemoglobin across all surveys was 1.3% and a median of 10% of women were missing pregnancy status; among the surveys of children, a median of 2.0% of children were missing age and 0.2% were missing hemoglobin. Of the included surveys (n = 87), 75 had both child and women data, seven had only child data, and five had only women data. Approximately equal numbers of surveys were conducted in each year– 21 in 2013, 25 in 2014, 19 in 2015, and 17 in 2016. These 87 surveys were conducted in camps in nine different countries, of which 41 (47.1%) were conducted in Chad and 18 (20.7%) were conducted in Kenya. Included among the 87 surveys were over 22,000 non-pregnant women aged 15 to 49 and over 45,000 children aged 6 to 59 months. Among the surveys of all women, the median average age of non-pregnant women was 28 [IQR: 27.2–28.7]. For children, the median average age across the surveys was 29.3 months [IQR: 28.1–31.1] and 50.7% were male.

**Table 1:** Distribution of cluster surveys by location of camp surveyed and year by target population.

	Total Cluster Surveys Conducted per Target Population		Total Surveys with both women and children	Total Unique Cluster Surveys
Location of Camp Surveyed^1^	Children	Women		
**Cameroon**	9	8	8	9
**Chad**	38	41	38	41
**DRC**	2	0	0	2
**Jordan**	2	2	2	2
**Kenya**	17	18	17	18
**Mauritania**	2	2	1	3
**South Sudan**	6	4	4	6
**Tanzania**	4	4	4	4
**Yemen**	2	1	1	2
**Total**	82	80	75	87

Descriptive parameters are presented in Table 2. Median sample size for children was approximately double the sample size for women (536.5 versus 260.5, respectively, Wilcoxon Signed Rank p<0.0001). Median anemia prevalence was higher in children than in women (42.3% versus 23.6%, respectively, p<0.0001). Survey-level means and standard deviations (SD) of the underlying distribution of hemoglobin values were significantly different between surveys of children and women, although the magnitudes of the differences were small, with a median hemoglobin mean of 11.1 g/dL and a median SD of 1.3 g/dL in children versus 12.8 g/dL and 1.5 g/dL in women, respectively (both p<0.0001). The majority of surveys had between 30 and 33 clusters, and no survey had fewer than 25 clusters.

**Table 2:** Survey-level distribution of sample sizes, anemia prevalence, and hemoglobin standard deviations for non-pregnant women and young children two-stage, cluster surveys.

Population	Median	IQR	Range
*Children*, *6–59 months (N = 82)*			
Sample Size	536.5	483.2–604.5	291.0–946.0
Number of Clusters	30.0	30.0–33.0	25.0–66.0
Mean Cluster Size	17.1	14.6–19.3	8.3–25.6
Anemia Prevalence	42.3	35.7–47.9	24.7–78.4
Mean Hemoglobin (g/dL)	11.1	10.9–11.3	9.6–11.9
Hemoglobin SD (g/dL)	1.3	1.3–1.4	1.1–1.7
*Non-pregnant women*, *15–49 (N = 80)*			
Sample Size	260.5	239.8–330.0	126.0–668.0
Number of Clusters	30.0	30.0–32.5	25.0–42.0
Mean Cluster Size	8.4	7.7–9.3	4.0–18.1
Anemia Prevalence	23.6	17.9–32.1	9.9–57.3
Mean Hemoglobin (g/dL)	12.8	12.5–13.2	11.7–13.8
Hemoglobin SD (g/dL)	1.5	1.3–1.6	1.2–2.0

SD: Standard deviation; IQR: Interquartile range.

### Distribution of design effects

**Fig 1:**
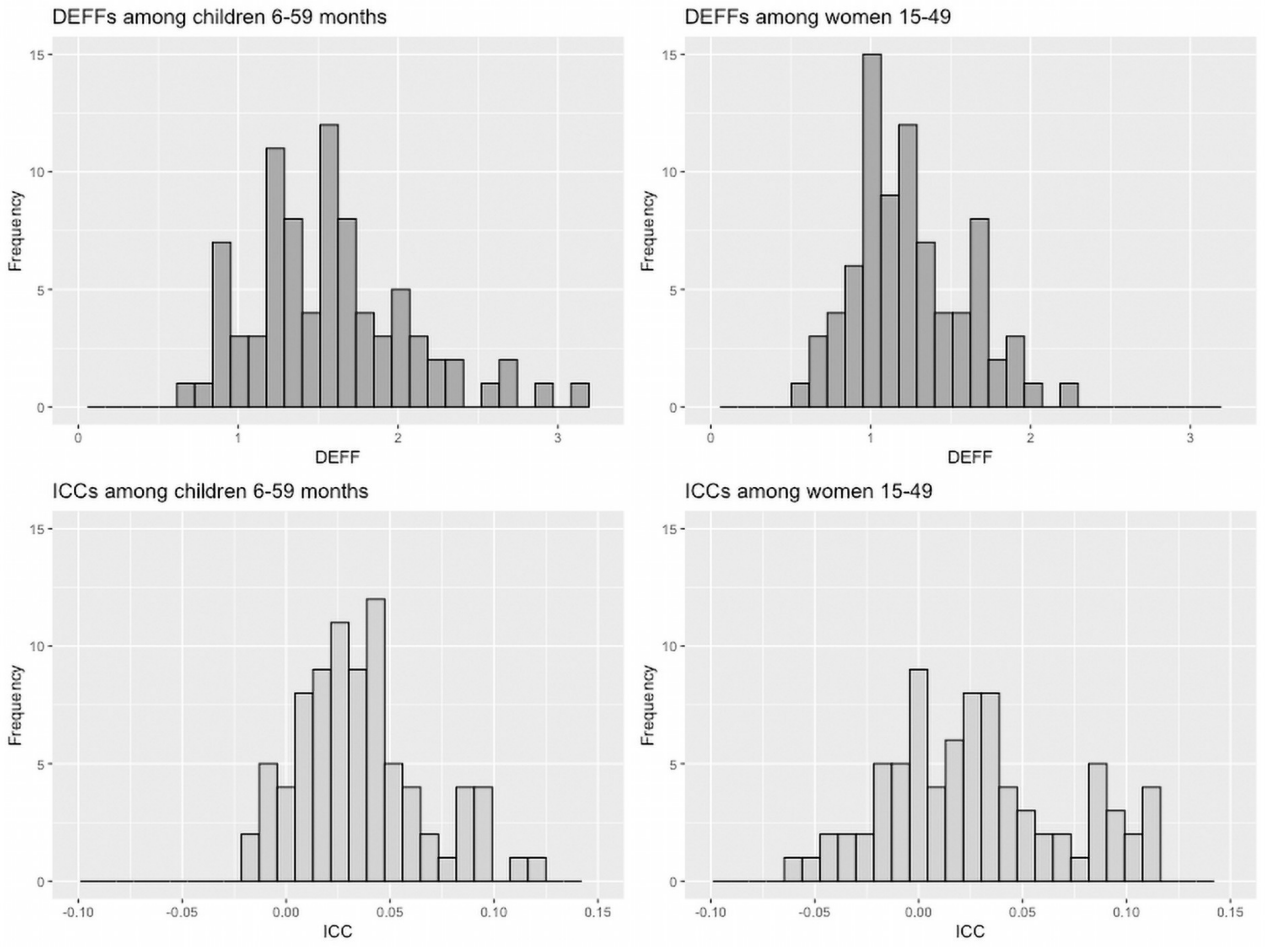
Distribution of anemia design effects and intracluster correlation coefficients for women and young children. DEFF: Design Effect; ICC: Intracluster correlation coefficient.

Histograms showing distribution of anemia DEFFs and ICCs for surveys of women and children are presented in Fig 1. Summary statistics to describe these distributions are presented in Table 3. The median DEFF for children 6–59 months was 1.54 (IQR 1.21–1.82) while for women it was 1.20 (IQR: 0.99–1.46), significantly smaller than for children, assessed via Wilcoxon signed-rank tests (p<0.001). Seventy-one percent of surveys of children and 91% of surveys of women had anemia DEFFs less than 1.75. (Table 3).

**Table 3:** Distribution of anemia design effects and intracluster correlation coefficients for women and young children.

	DEFF						ICC					
	Median	IQR	Range	% DEFF under 1.50	% DEFF under 1.75	% DEFF under 2.00	Median	IQR	Range	% ICC under 0.020	% ICC under 0.050	% ICC under 0.100
**Children**	1.54	1.21–1.82	0.72–3.19	45.1%	70.7%	82.9%	0.032	0.015–0.048	-0.019–0.120	32.9%	76.8%	97.6%
**Women**	1.20	0.99–1.46	0.58–2.20	76.3%	91.3%	97.5%	0.024	-0.002–0.055	-0.060–0.145	45.0%	72.5%	91.3%

DEFF: Design effect; ICC: Intracluster correlation coefficient; SD: Standard deviation; IQR: Interquartile range.

In contrast to DEFF, median ICCs were not significantly different between women and children (p = 0.17). The median ICC for children was 0.032 (IQR: 0.015–0.048) and for women the median was 0.024 (IQR: -0.002–0.055). For both populations, approximately three-quarters of ICCs fell below 0.05 and more than 90% were below 0.10 (Table 3).

### Correlations

Spearman’s correlations of DEFF and ICC for the 75 paired surveys (which measured hemoglobin in both women and children in the same households) was low and insignificant for DEFF [0.11 (p = 0.344)] and for ICC [0.18 (p = 0.112)].

### Assessment of variability by region

The overall one-way Kruskal Wallis test investigating differences in DEFF and ICC by country or region in surveys with both women and children studies revealed significant differences in DEFF and ICC (p = 0.006 and p = 0.018, respectively). Dunn’s test pairwise differences by region showed that Kenya had significantly higher DEFFs and ICCs when compared to Chad. For ICC, other countries grouped together were significantly larger than Chad (Table 4). Mean cluster size was significantly different by region (p = 0.026), with larger median cluster sizes in Kenya (13.2 [IQR: 10.8–20.7]) versus Chad (10.1 [IQR: 8.2–16.8], p = 0.015) or other countries combined (12.4 [IQR: 8.6–14.4], p = 0.042).

**Table 4:** Pairwise Dunn’s Test differences in ICC and DEFF by region.

	DEFF			ICC		
Overall	p = 0.006			p = 0.018		
	**Median (IQR) Chad**	**Median (IQR) Kenya**	**p-value**	**Median (IQR) Chad**	**Median (IQR) Kenya**	**p-value**
**Chad vs. Kenya**	1.27 (0.98–1.55)	1.60 (1.21–1.94)	0.003	0.024 (-0.003–0.042)	0.037 (0.020–0.062)	0.022
	**Median (IQR) Chad**	**Median (IQR) Other**	**p-value**	**Median (IQR) Chad**	**Median (IQR) Other**	**p-value**
**Chad vs. Other**	1.27 (0.98–1.55)	1.33 (1.16–1.72)	0.140	0.024 (-0.003–0.042)	0.029 (0.013–0.085)	0.048
	**Median (IQR) Kenya**	**Median (IQR) Other**	**p-value**	**Median (IQR) Kenya**	**Median (IQR) Other**	**p-value**
**Kenya vs. Other**	1.60 (1.21–1.94)	1.33 (1.16–1.72)	0.253	0.037 (0.020–0.062)	0.029 (0.013–0.085)	1.00

Comparisons with p<0.05 considered statistically significant.

## Discussion

Increased attention to micronutrient deficiencies among refugees, and the resulting interest in monitoring population prevalence of anemia, necessitates improved evidence on anemia design parameters to inform sample size calculations for cluster surveys. This is the first comprehensive analysis of anemia DEFFs, providing evidence aimed at improving efficiency of household surveys to assess anemia in refugees and other populations.

Presented analysis demonstrates that ICC of anemia is relatively small for both women and children–on the magnitude of approximately 0.03. Small ICCs suggest that prevalence of anemia can be estimated efficiently in a survey with a cluster design, a contrast to other household-level indicators that have been shown to have markedly higher ICC values (>0.3) [13]. Notably, ICCs were not significantly different comparing surveys of non-pregnant women and children suggesting that these two target populations have similar heterogeneity of intra-cluster distributions. DEFFs were significantly higher for children than for women. However, these differences are a function of sampling design (i.e., different average cluster sizes) rather than underlying differences in the population distribution of anemia cases. Surveys assessed generally had larger mean cluster sizes for children. Given ICCs in the range of 0.02 to 0.04, surveys with an average of 10 eligible individuals per cluster should expect DEFFs in the range of 1.2 to 1.4 whereas surveys with an average of 20 eligible individuals per cluster should expect DEFFs in the range of 1.4 to 1.8. In the absence of other information, using a DEFF of 1.50 should be large enough for surveys of women given a median mean cluster size of 8.4, while a DEFF of 1.75 for children would be more appropriate given median mean cluster size of 17.1. In locations surveying both women and children, DEFFs for the larger mean cluster size should be chosen. However, regional differences are notable, including larger ICCs and DEFFs in Kenya; similarly, larger cluster sizes would warrant larger DEFFs. Field guidance for calculation of sample size to achieve adequate precision of anemia estimates can be simplified to relate to the number of eligible individuals per cluster, to be applied equally to women and children.

Paired analyses, assessing the subset of surveys that measured hemoglobin among both target groups in selected households, provides evidence of poor correlations between the DEFFs and ICCs for anemia prevalence in women and that of children, suggesting that values observed in one target group cannot be used to predict values in the other.

Country specific analyses are presented to explore potential geographic differences in clustering of anemia prevalence. Kruskal-Wallis test of the paired results suggest significantly lower ICCs for surveys conducted in Chad relative to Kenya (p = 0.022) and the other seven countries (p = 0.048). Whether meaningful geographic differences exist in other contexts will need to be explored with more data from additional countries. Given that these regional patterns persist in ICC, not only DEFF, this suggests a stronger regional difference beyond differing cluster sizes, despite significantly larger mean cluster sizes in Kenya.

There are several limitations to our analyses. First, all surveys were conducted among refugee populations. We note that the observed hemoglobin distributions in these cluster surveys is consistent with findings from national surveys, where a recent analysis of 80 Demographic and Health (DHS) surveys found a mean hemoglobin SD of 1.48 for children aged 6–59 months and 1.58 among non-pregnant women aged 15–49 [26]. However, confirmation of the generalizability of these findings to non-displaced populations requires further analysis of national surveys. Second, the majority of the surveys including anemia conducted by UNHCR were of refugees in Chad and Kenya. Variability in the nine other countries, included in the analyses as one group, may not be well captured given the small sample. Third, we had a relatively small number of surveys among women and children in the same camp, and more data are needed to confirm these findings.

## Conclusions

This manuscript adds to the very limited evidence on design effects and intra-cluster correlation coefficients of anemia prevalence in cluster surveys, an important nutrition indicator highlighted among the six nutrition targets for 2025 by the 2012 World Health Assembly. We identify a DEFF of 1.75 to be sufficient for surveys of 20 women or children in the absence of other information for guiding sample size calculations. These findings support current UNHCR field guidance in simplified sample size calculation and provide evidence to inform efficient survey design to ensure adequate precision for anemia assessments in refugees, and possibly populations more generally.

## Supporting information

S1 File (CSV)

## Abbreviations

CDC: U.S. Centers for Disease Control and Prevention
CI: Confidence Interval
DEFF: Design Effect
DHS: Demographics and Health Survey
ICC: Intracluster Correlation Coefficient
IQR: Interquartile Range
SD: Standard deviation
SENS: Standardised Expanded Nutrition Survey
SMART: Standardized Monitoring and Assessment of Relief and Transitions
UNHCR: United Nations High Commissioner for Refugees
WHO: World Health Organization
